# *The Veterinary Quarterly* 2017 JCR impact factor increased from 1.176 to 1.492

**DOI:** 10.1080/01652176.2018.1550852

**Published:** 2019-02-18

**Authors:** J.H. van der Kolk, Merran Govendir

**Affiliations:** a Swiss Institute for Equine Medicine (ISME), Vetsuisse Faculty, University of Bern, Bern, Switzerland;; b Sydney School of Veterinary Science, The University of Sydney, Sydney, Australia

*Dear reader,*

It is our pleasure to announce that *the Veterinary Quarterly* has seen its 2017 JCR Impact Factor increase from 1.176 to 1.492. As a consequence, it has also moved up in the rankings, and is now 41/140 in the Veterinary Sciences JCR Category (previously 47/136).

*The Veterinary Quarterly* is an international open access journal which publishes review articles and original research in the field of veterinary science and animal diseases. As such the manuscript entitled ‘*Trueperella pyogenes* multispecies infections in domestic animals: a retrospective study of 144 cases’ by Ribeiro et al. (2015) is fully within the scope of the journal. The authors of this manuscript from the Sao Paulo State University in Brazil are to be congratulated as their manuscript is the top-cited article within the 2015 volume.

The top-cited article within the 2016 volume was by Zorriehzahra et al. entitled ‘Probiotics as beneficial microbes in aquaculture: an update on their multiple modes of action: a review’. The group led by Dr Dhama from the Indian Veterinary Research Institute, Izatnagar, Bareilly in India is to be congratulated with this success too.

Dr Dhama not only is a very appreciated editorial board member of *the Veterinary Quarterly*, but he has been identified as a hyperprolific author (one out of 265) by Nature (Ioannidis et al. 2018). Hyperprolific authors are defined as authors who had published more than 72 papers (the equivalent of one paper every 5 d) in any one calendar year between 2000 and 2016. Dr Dhama started to publish for *the Veterinary Quarterly* in 2014 and till to date he has published 15 papers in total for *the Veterinary Quarterly*. This effort by Dr Dhama and his excellent team is greatly acknowledged and certainly contributed substantially to the increase in the JCR Impact Factor of *the Veterinary Quarterly* over the recent years.

Last but not least, the increase in *the Veterinary Quarterly* JCR Impact Factor largely attributed to the great support from the authors, reviewers and editorial board members. This is a good opportunity for Merran and myself to sincerely thank all *Veterinary Quarterly* reviewers and editorial board members for their thorough and timely comments that always improve the submitted manuscripts as well as the administration and editing teams at Taylor and Francis, the journal’s publisher, also providing excellent support to the Editorial Board.

With the authors’ and reviewers’ assistance, *the Veterinary Quarterly* looks forward to increasing its Impact factor even higher in future years. Perhaps best to keep Dr Dhama’s advice in mind to persue ‘with a high team spirit, following very hard works and a passion to serve science’ (Supplementary text page 34 to Ioannidis et al. 2018).

J.H van der Kolk *Swiss Institute for Equine Medicine (ISME), Vetsuisse Faculty, University of Bern, Bern, Switzerland*
johannes.vanderkolk@vetsuisse.unibe.ch
Merran Govendir *Sydney School of Veterinary Science, The University of Sydney, Sydney, Australia*
merran.govendir@sydney.edu.au

